# Environmental considerations in human genomic data governance: overcoming normative challenges

**DOI:** 10.1186/s12910-026-01417-3

**Published:** 2026-02-24

**Authors:** Narcyz Ghinea, Wendy Xin, Gabrielle Samuel, Anneke Lucassen, Ainsley J. Newson

**Affiliations:** 1 Sydney Health Ethics, Sydney School of Public Health, Faculty of Medicine and Health, University of Sydney, Sydney, Australia; 2 Ethics and Agency Research Centre, School of Humanities, Faculty of Arts, Macquarie University, Sydney, Australia; 3 Department of Global Health and Social Medicine, King’s College London, London, United Kingdom; 4 Centre for Personalised Medicine, Nuffield Department of Medicine, Oxford University, Oxford, United Kingdom

**Keywords:** Environment, Genomics, Big data, Ethical issues, Governance, Data management, Innovation

## Abstract

**Background:**

Human genomics involves generating, processing, storing, using and sharing immense amounts of information. This will only increase given efforts to embed whole genome sequencing into mainstream clinical practice. However, as society becomes more conscious about the impact of human activity on the environment, it is imperative to consider what this means for the future of genomic data generation and its governance.

**Methods:**

There is scant literature on environmental ethics in the context of genomic data governance. To address this gap, we drew on existing debates and scholarship relating to the environmental impact of biobanking, healthcare generally, and data-intensive healthcare specifically, to construct the strongest conceptual challenges to the proposal that environmental concerns should be incorporated into genomic data governance frameworks. Each challenge was critically examined.

**Results:**

We developed five conceptual obstacles to incorporating environmental considerations in genomic data governance: genomic data optimism; the outcome measurement challenge; the responsibility challenge; the worse offender challenge; and technological idealism. We argue that no objection, when considered individually or together, provides strong enough reasons to ignore or de-prioritise environmental considerations in genomic data governance. We suggest three proposals to move forward with environmental ethics in genomic data governance: research ethics committees should pilot how to consider environmental ethics when assessing and approving genomic research; environmental considerations should be explicitly considered in all prospective genomic data generation; the sector should catalogue low-value practices that can be easily discontinued with minimal disruption.

**Conclusion:**

As a rapidly growing field, the environmental harm from genomic data generation is certain and significant. The relevant question, then, is not whether but how to mitigate the harms without compromising genomics’ potential benefits.

## Background

Deploying genomic sequencing in human health is a data-intensive enterprise. While genetic tests were once targeted at a particular gene or genes thought to be relevant to a person’s health or family history, technological advances in DNA sequencing have effectively reversed this approach and genomic tests increasingly cover the whole genome to identify patient-relevant results. This disruptive change is driven by laudable aims, including generating new knowledge, increasing diversity within datasets, and offering diagnoses or treatments to patients and families with rare or undiagnosed conditions.^1^ Nevertheless, while the potential health benefits of human genomic data are significant, the data infrastructures that are necessary to support its generation and storage come with inevitable environmental costs [1]. In this paper we wish to temper the momentum of genomic data generation and storage to consider the environmental ethics involved.

With the ever faster, cheaper, and more accessible nature of sequencing, the amount of genomic data collected, processed, analysed and stored is growing rapidly. It is estimated that all the human genomic data generated to date has required up to 40 exabytes of memory [2], the equivalent of over 80 million 512GB laptops. Further, the Global Alliance for Genomics and Health state that genomics is comparable to YouTube, astronomy, or particle physics in terms of data demands [3]. While there is scant empirical evidence demonstrating how genomics will compare with other health technologies (e.g. MRIs, CTs, etc.) in terms of data demands on a global scale, it is clear that genomic data is a category of big data with significant resource demands, and that its environmental impact is substantial in its own right [2]. This status raises critical ethical questions regarding whether these impacts are justified and whether the ongoing trend of collecting and storing increasing amounts of genomic data is always ethically defensible.

To our knowledge, there is no environmental (bio)ethics literature that specifically addresses genomic data. In this paper, we present five key normative challenges to tackling the environmental impact of genomic data that bioethicists must address: genomic data optimism; the outcome measurement problem; the responsibility challenge; the worse offender challenge; and technological idealism. We critique each in turn and argue that none of these pose an insurmountable barrier to taking environmental considerations seriously in human genomics.

## Methods

Normative bioethics analyses involve analytic reasoning and the use of tools such as conceptual clarification and argumentation with the goal to develop, evaluate, and refine moral claims and principles [4, 5]. In this paper, we specifically focused on identifying normative considerations that could develop the strongest possible challenges to incorporating environmental considerations in a genomic data governance framework. The authors (who have expertise in bioethics, genomics, and environmental impact of healthcare) critically examined each challenge in turn to determine whether they provide defensible grounds for omitting environmental considerations from genomic data governance.

## Results

We developed and critically examined five broad conceptual challenges to the claim that environmental considerations need to be taken seriously by individuals working in the human genomics sector. An overview of these challenges is provided in Table 1.

**Table 1:** Main conceptual challenges to incorporating environmental ethics in genomic data governance

Conceptual challenge	Points that potentially defeat the challenge	Compromise
Genomic data optimism: *Collecting and analysing larger datasets will enable medical progress.*	• More data than necessary is routinely collected. • Significant ‘dark data’: lost and not managed. • Knowledge gap: much genomic data is not understood and not usable. • Promissory value: many of the benefits haven’t yet been realised.	Identify low-value data practices and curtail them. Can’t uncritically accept promissory value of genomic data.
The outcome measurement challenge: *We can’t measure environmental harms reliably and so can’t account for them.*	• Certainty of environmental harms. • Similar inability to reliably measure benefits of genomic data. • Ethics is not limited to quantifiable considerations. • Focus on measurable outcomes can lead to bias.	Measurement can provide a clearer understanding of what is at stake, but doesn’t determine ethical debate.
The responsibility challenge: *It is difficult to assign responsibility to individuals.*	• All can and should choose the available option that is the least worst for the environment. • Can identify specific green strategies that impose a duty under limited circumstances.	Impose only a weak duty to choose the least environmentally worst option reasonably available. Institutions should incorporate epistemic and control conditions that enable environmental choices. Strong duty could be imposed in the context of targeted initiatives.
The worse offender challenge: *Other industries are less valorous yet more polluting.*	• Environmental harm is cumulative, and the response also needs to be. • Benefits of genomics may accrue to the few, but environmental harms impact all. • Many other major polluting industries would be excused undermining any environmental action.	No compromise. Challenge doesn’t hold.
Technological idealism: *Greener technologies will solve the problem.*	• Rebound effect – greater efficiencies (e.g. energy efficiency/ cheaper cost) lead to more intensive utilisation mitigating any advantages.	Any gains realised by technology must be distributed by behavioural interventions guided by ethical deliberation.

### The genomic data optimism challenge

In data-intensive healthcare, there appears to be an implicit assumption that collecting and analysing ever larger datasets is a public good [6, 7]. There is no doubt that genomic data has helped avoid lengthy diagnostic delays and created novel treatment opportunities. However, this doesn’t mean all genomic data generated and stored is currently or even imminently useful. Intraspecies genomic variations are incredibly small, with humans sharing approximately 99.9% of their genomes with each other [8]. Additionally, our ability to use, interpret and apply information arising from stored genomic data remains limited. Significant knowledge gaps remain regarding gene-gene interactions, gene-environment interactions, variant-phenotype relationships, ancestral diversity, and the role of non-coding regions [9, 10]. Even referring to these as ‘gaps’ is misleading. Genomic variation depends on stochastic factors that may make it impossible to ever have complete information about what genomic variants do. Thus, there remains much to understand regarding how well the genome predicts diseases, or their successful treatments. Despite some successes in rare diseases, for most conditions the predictions are too weak to be of clinical utility. Moreover, for complex conditions especially, genomic information will only ever be part of the explanation for the disease [11, 12]. 

Further complications are raised by the fact that not all genome sequencing is equal. Sequencing technologies have become significantly more accurate, with recent methods showing orders of magnitude fewer errors per base [13, 14]. This suggests that at least some parts of genomes sequenced in the past may become (or may already be) redundant, raising questions about the value of long-term storage of at least some data. Also, as more genomic data is collected in hopes of gaining new insights, much of it may become “dark data” - never used nor managed, but still consuming energy [15]. A possible counter to this view is that it is worth keeping genomic data that is currently poorly understood, as it may become valuable in time as knowledge advances. However, this promissory value of keeping data “just in case” must be considered against its environmental implications.

If we are to take environmental concerns arising from human genomics seriously, it would be reasonable to propose that we should, at the very least, avoid collecting and continuing to store low-value genomic data. This includes data that duplicates existing datasets, is now considered to be of lower quality (e.g. higher error rates), lacks interpretive clarity, has unclear research or clinical value, or is stored without a defined future use [16]. It is beyond the scope of this paper to define low-value data in detail, but it seems clear that large amounts of genomic data are potentially disposable, or should not be obtained in the first place, without any significant adverse impact on intrinsic or translational knowledge. Given that there is now wide support for defining and curtailing low-value healthcare more generally, it is consistent to undertake similar activity in health genomics^2^, including examining the storage of data that supports it [17]. This involves not only deciding what data to store, but what mix of data are needed to realise health benefits [18]. 

Overall, in response to the genomic data optimism challenge, it seems reasonable to conclude that not all human genomic data that is generated and stored is valuable. Genomic data has an environmental impact, and this impact should be explicitly considered. One way to do this is by prospectively using data storage formats like Compressed Reference-oriented Alignment Map [19], which only stores genomic variants relative to a reference sequence. We must move beyond an uncritical acceptance of the promissory and hopeful discourse that underpins genomics, and which justifies the collection, storage, and processing of ever greater amounts of data.

### The outcome measurement challenge

A second normative challenge to incorporating environmental harm into decision-making on genomic data generation and storage is ambiguity around measuring harms from these processes [20]. The measurement challenge has also arisen in biobanking,^3^ where lack of information and lack of methods are barriers to incorporating environmental considerations [21]. In genomic data activities, not only are environmental harms challenging to measure, so are benefits, especially in research where benefits are inherently uncertain [22, 23]. It could therefore be argued that it is too difficult to incorporate environmental considerations into our ethical calculus.

But even if we accept measurement challenges, we don’t believe they negate an ethical requirement to consider the environmental impacts of genomic data generation and storage. We still must weigh up ultimately incommensurable concerns. For instance, healthcare systems routinely use sophisticated methods to balance quality of life improvements from new interventions against economic considerations. Nevertheless, what health systems are ultimately willing to pay is (often controversially) a matter of values and priorities rather than pure rational deduction [24]. Similarly, even if we could accurately measure the benefits of genomics and its environmental harms, we would still be left with the challenge of deciding how to value one relative to the other.

Additionally, bioethics provides us with the tools to inform what we should do, regardless of whether an activity can be quantified. This is ultimately a reflection of what we value as a society. If we were to demand that ethical deliberation is only meaningful in the context of accurately measured benefits and harms, we would also be inadvertently proposing that it is not possible to attribute ethically justified responsibilities for activities that cannot be measured. This is clearly unreasonable, as we routinely make ethical demands of each other based on values and morals, not merely quantifiable consequences. Additionally, if one were to only account for what can be measured, it would bias our decision-making towards concerns that are easily measurable. This can lead to false standards of success, veil a deeper understanding of issues, and undermine integrity [25]. 

Therefore, while measurement can no doubt help decision-makers better understand what is at stake, good and ethical decision-making is not dependent on it. To the contrary, ethical decisions must be sensitive to how measurement can bias our decisions and potentially undermine important social goals and public goods.

### The responsibility challenge

In 2022, the International Code of Medical Ethics introduced the general principle that doctors should consider environmental sustainability in their practice [26]. This reflects scholarship in green bioethics which has proposed, *inter alia*, that: doctors should, where possible, prescribe less environmentally harmful treatments; [27] doctors should inform patients and gain informed consent regarding environmental harms of treatments; [28, 29] and that doctors should adopt environmental activism as part of their identity [30]. Additionally, the Planetary Health movement calls upon public health practitioners to act as the “independent conscience of planetary health” [31]. However, the ethical basis for assigning such broad responsibilities to health professionals remains tentative [32]. When it comes to human genomics, there are many actors involved, such as data custodians, laboratory scientists, bioinformaticians and so on, and it is unclear how to divide responsibilities for environmental concerns between them, if they are even aware of such concerns. And as we discuss below, the environmental consequences of genomic data are epistemically challenging to conceive of.

That certain activities lead to environmental harm does not necessarily mean those who perform these activities are to blame and are therefore accountable. This is particularly true if the harms are not a primary outcome of the activity but rather a potentially uncontrollable downstream consequence. If we were to assign responsibility for all downstream harms to individuals, it would be challenging to delimit our obligations for a multitude of secondary outcomes beyond our immediate control or awareness.

To address this in the context of health care, it has been argued that there should be a clear distinction between doctors’ obligations and aspirations, where obligations relate to factors that have a direct impact on health (which is doctors’ primary professional role) and that they can directly modify. On this basis, it has been proposed that doctors are not responsible for considering broad socioeconomic concerns that may impact health, and by extension, we could say that they have even less responsibility for environmental concerns [33]. 4 It has also been posited that shifting the focus of medicine from patient-centric to socio-centric goals would undermine its telos and lead to the medical professional becoming instruments for political ends [34]. 

These criticisms are generally applicable to any context where we ask professionals to take responsibility for factors that lie outside of their primary role and function. One way to address this is to propose only a weak duty, where environmental considerations are recognised as important but secondary to one’s primary role. In this case, individuals with a choice between similarly appropriate options, but where there is a significant divergence in environmental impact, are responsible for selecting the option that produces the least harm. This principle is implicit in Parker’s argument proposing that less polluting asthma inhalers should be prescribed by physicians as a matter of course [27]. 

However, to extend such duties to a wider context demands we have easy access to information about the relative environmental harms of options, and as we have noted above, such information may not be easy to attain. An entirely new epistemic infrastructure would need to be developed to ensure this information is easily available to those assigned responsibility. Otherwise, gaining the requisite knowledge may be too burdensome, violating the reasonability criterion attached to duties and thereby absolving agents of responsibility [35]. 

Alternatively, we could attribute responsibility to individuals or groups only for specific practices that are known to have an environmental impact, rather than environmental harm generally, as per Parker’s asthma inhaler proposal [27], or projects such as the Green Anesthesia Initiative [36]. 

In summary, we recognize that assigning a strong duty upon individuals in contexts where they have little control or insufficient information (or understanding) to enact the duty is not feasible. However, we believe there is still scope for individuals to choose the least-worst environmental option among feasible options known to them. We also believe institutions have a responsibility to guide and support individuals by providing the control and epistemic conditions to enact environmental duties in the human genomics ecosystem. This can include introducing initiatives to minimise environmental harm, to encourage consideration of environmental impact in research ethics review and funding decisions, and to educate on environmentally responsible practices [37]. 

### The worse offender challenge

In biobanking, an argument against taking environmental considerations seriously is that the large environmental impact of other activities with less altruistic goals should be the target of action first [6]. More generally, it has also been argued that healthcare and related research is exceptional as a whole due to its noble aims [38]. This line of argument implicitly calls for the prioritisation of non-health activities for environmental action. If this holds true, then health-related genomic data activities should be exempted or de-prioritised for environmentally driven constraints.

However, if we accept this argument, we should also delay assessing the environmental impact of all activities with similar or even more noble aims than human genomics. For instance, we would have to consider whether to deprioritise the environmental impact of all medical research, as well as all agriculture and food production, and so on [39]. Perversely, this would undermine a significant opportunity to tackle environmental harm.

Additionally, it is uncertain whether we can in fact prioritise activities so simply. For instance, it has been proposed that globally, modern healthcare and all processes connected to it primarily benefit those who are well-off. Yet the environmental harms generated by healthcare impact everyone, and disproportionately those living in low or middle-income settings [40]. Therefore, what is deemed a high-priority activity for the few that disproportionately benefit would not be so for the majority. This unfair distribution of benefits and environmental harms is a key focus of the green bioethics movement [40]. 

Since it is our cumulative actions and decisions over a prolonged period rather than any specific action that causes environmental harm, then cumulative action is also the primary means of mitigating the harm. From this standpoint, all individuals should avoid actions that they know or expect to cause environmental harm, or avoid being complicit in such actions, whenever possible, regardless of their extent [41–43]. If we accept this understanding, then which industry is the ‘worse offender’ no longer matters, as the justification for mitigating environmental harm is not associated with the goals of the offending activities, nor the magnitude of harm caused by that activity. Rather, it is premised on the existence of a feasible opportunity to minimise environmental harm, regardless of context [44]. 

### Technological idealism

Another argument against the need to consider environmental impact in human genomics is that greener technology, algorithms, and infrastructure could mitigate any concerns. For instance, in biobanking, more energy-efficient freezers, better insulation, and real-time monitoring of samples are raised as ways to minimize the environmental footprint [45, 46]. In the context of big data and artificial intelligence, more efficient algorithms, optimising functionality to reduce waste, and utilising green energy have been explored [47]. In relation to genomic data, there have been attempts to enhance efficiency of systems which also would positively impact environmental sustainability, such as data storage formats that only store variants from a reference genome rather than an entire genome, and compression techniques that reduce data storage requirements of laboratories [48]. 

The primary argument against relying solely on such advances to address environmental challenges is that energy efficiency gains which may at first seem to address our environmental concerns can in fact be the *cause* of more intensive utilisation that ultimately undermines their advantages, or at the very least, fail to realize its full benefits. In other words, efficiency claims can perversely drive more uptake and use, just as building more and bigger roads might motivate more people to use cars. While there is debate about its magnitude and importance, there is a general acceptance that such an effect exists, known as the ‘rebound effect’ [49, 50]. 

In the context of genomic data, the rebound effect may play out as follows. A new data format promises to reduce the environmental footprint of storage by requiring only a fraction of an entire genome to be stored, and a new compression technology enables this data to be stored on far less digital space. Users across the genomic data ecosystem are keen to adopt the technology as it reduces the cost of storage and makes the data easier to share. This could in turn lead to more intensive sharing and utilisation of the data, leading to increased energy consumption. Additionally, as data becomes easier and cheaper to generate, process, store and share, this trend may lead to more low-value research, as less attention is given to weighing its costs and benefits. As noted in the context of data-driven precision medicine initiatives, more data, and more easily accessible data, allow researchers to use ever more “powerful (and energy hungry) algorithms to answer endless health-related research questions” [7]. 

The relationship between energy efficiency gains and greater overall energy consumption suggests that the implementation of greener technologies and processes is insufficient to address environmental harms. At some point, we must intervene in the market and make moral judgements and place restrictions around how much genomic data to generate, what types to keep, and how this data is to be utilised most effectively [18, 51]. Such restriction could be achieved in various ways, for example through direct restrictions, or subtler means such as nudging, with the best approach depending on context. Therefore, while we cannot ignore the important role that technology will play in environmental sustainability, we also cannot assume or trust that it will solve the problem.

## Discussion

In this paper, we have sought to contribute to a growing body of literature on environmental and green bioethics by exploring whether it is feasible and reasonable to consider environmental concerns in human genomic data generation and storage. This work contributes to a larger project to inform genomic data governance [52]. 

We have approached this issue by explicitly addressing what we see as the most significant normative challenges to incorporating environmental considerations regarding genomic data. Our analysis demonstrates that these challenges do not defeat a general responsibility to incorporate environmental considerations in genomics. The real barriers are feasibility and lack of awareness. In this article, we did not address the structural and cultural factors that also undermine environmental sustainability, yet they are also important to consider. This has been explored by other authors in related contexts such as biobanking, which has shown commercialisation and privatization, a competitive operating environment, fragmentation of the industry, data security, privacy, and consent, are all barriers to instituting more environmentally friendly practices [16, 53]. 

To address feasibility concerns, we believe that it is necessary to recommend actions that are not excessively burdensome to the genomics community. We propose that Human Research Ethics Committees should pilot environmental ethics in their deliberations, to see how it can be implemented in the real world, especially as it relates to genomic data retention and management which is a key consideration in ethics applications [37]. This could be the basis for the development of empirically informed future guidelines that are more broadly applicable to the wider health genomics community. Second, we believe it is possible for the genomics community to work together now to identify specific practices that are low-value yet contribute to environmental harm and agree to discontinue them just as others elsewhere in the health community are currently doing. For instance, there could be agreement that all genomic sequence data that would now be considered of inadequate read depth, or that is “locked up” due to consent demands and unlikely to be usable, be deleted or reduced unless there are strong justifications for keeping it. There are also a variety of prospective changes that can be made, including giving consideration to data storage formats, and routinely considering whether data needs to be generated in the first place (and if so, what data should be processed and stored).

There is also a need to raise awareness of this issue among all actors within health genomics. This is particularly important at this time given ongoing activity to translate genomics into everyday health care and preventive health, which will exacerbate its impact. Bioethicists have an important role to play in bringing to light the promissory value of human genomics while also being sensitive to pressing environmental concerns.

## Conclusion

Until now, little scholarship in bioethics has explicitly addressed the environmental impact of genomic data. In this paper, we have identified and critically assessed five key normative challenges to implementing environmental ethics in genomics: genomic data optimism; the outcome measurement problem; the responsibility challenge; the worse offender challenge; and technological idealism. We argue that none of these provide strong reasons, either as stand-alone considerations or collectively, to ignore environmental considerations when it comes to genomic data generation, processing, storage, use and sharing. As a result, all actors working with, or regulating, genomic data have an individual and collective responsibility to heed the environment in their work. This should include recommending feasible steps that institutions, groups and individuals working in and with genomic data ecosystems can implement to improve its environmental credentials. In this regard, we have endorsed several proposals: that research ethics committees should pilot how to consider environmental ethics when assessing and approving genomic research, that environmental considerations should be explicitly considered in all prospective genomic data generation, and that the sector should catalogue low-value practices that can be easily discontinued with minimal disruption.

## Data Availability

Not applicable.

## Footnotes

1 Arguably a more contested aim is profit-generation for commercial entities through partnership with health providers. Consideration of this issue is beyond the scope of this paper.

2 Here we recognize that this will necessitate tackling some significant questions, including genetic determinism narratives, and politically or commercially motivated agendas that drive a ‘more is better than less’ narrative. These questions are important but are beyond the scope of this paper to address.

3 Biobanking is closely related with genomic data repositories in that genomic data is derived from bio-samples and major biobanks store genomic data with their samples.

4 This proposal raises interesting issues on the current focus of health on individuals, possibly edging out consideration of socioeconomic and structural factors. If specialisms such as public health doctors and general practitioners diminish in number, then this may provide further barriers to considering environmental concerns.
